# Developmental Language Disorder and Risk of Dyslexia—Can They Be Told Apart?

**DOI:** 10.3390/bs15091234

**Published:** 2025-09-10

**Authors:** Aliki Chalikia, Asimina M. Ralli, Faye Antoniou

**Affiliations:** 1 Department of Educational Studies, National and Kapodistrian University of Athens, 15772 Athens, Greece; fayeantoniou@eds.uoa.gr; 2 Department of Psychology, National and Kapodistrian University of Athens, 15772 Athens, Greece; asralli@psych.uoa.gr

**Keywords:** developmental language disorder, Dyslexia, phonological processing, verbal working memory

## Abstract

Developmental Language Disorder (DLD) and Dyslexia frequently co-occur. Many studies suggest that children with Dyslexia may also struggle with oral language skills, while those with DLD may also face challenges in word decoding. Both groups of children typically exhibit deficits in phonological processing [phonological awareness, rapid automatized naming (RAN), verbal short-term memory (VSTM)] and verbal working memory (VWM). Despite the increasing number of comparative studies, few have systematically examined these overlaps in children at the early stages of literacy acquisition, and few studies have systematically assessed all oral language subsystems. This study investigates: (a) differences and similarities among children with DLD, children at Risk of Dyslexia (RfD), and typically developing children (TD) in phonological processing (phonological awareness—implicit and explicit—VSTM, RAN), VWM, oral language, and word decoding; (b) patterns of performance across groups; and (c) correlations between phonological processing and VWM skills with oral language and word decoding. The participants were 45 first graders (Mage = 6.8), equally divided into three groups (DLD, RfD, and TD children). Both special groups (DLD, RfD) performed significantly worse than the TD group across nearly all measurements. DLD children showed pronounced oral language and VSTM deficits, while RfD children primarily struggled with decoding and explicit phonological awareness. Group-specific correlations emerged. The findings are discussed in light of the theoretical models of the relationship between DLD and Dyslexia taking into account the specific developmental stage.

## 1. Introduction

Developmental Language Disorder and Dyslexia are two neurodevelopmental disorders that can negatively impact school attainment and social interaction (Matte-Landry et al., 2020; Snow, 2016; Ralli et al., 2021b). Over the past two decades, increasing research interest has focused on the relationship between these two disorders, not only because both disorders frequently co-occur in the same individuals (McArthur et al., 2000) and show substantial overlaps in their underlying cognitive mechanisms, particularly in phonological processing (Snowling et al., 2019b; Ramus et al., 2013), but also because clarifying this relationship has important implications for diagnosis, intervention and prognosis (Bishop et al., 2017; Snowling & Hulme, 2012; Adlof, 2020).

Both disorders are thought to reflect broader neurocognitive constraints, including deficits in general-domain processes such as statistical learning, implicit learning, and memory (Evans et al., 2009; Arciuli & Torkildsen, 2012; Lallier & Valdois, 2012). While much research has focused on shared phonological processing (phonological awareness, RAN, verbal short-term memory (VSTM)), relatively few studies have examined verbal working memory (VWM) (e.g., Alloway et al., 2017) despite strong evidence suggesting that poor VWM may constrain linguistic skills in children with DLD (Vugs et al., 2016; Ralli et al., 2021a). Similarly, implicit phonological awareness, which is associated with vocabulary development (Metsala & Walley, 2013), has not been included in most comparative studies. Moreover, most existing studies lack comprehensive assessments of all oral language components (e.g., Bishop et al., 2009). As a result, the broader oral language profile of children with Dyslexia—especially in comparison to children with DLD—often remains underinvestigated (Guasti et al., 2015). Also, most of the existing studies have recruited either preschool children (e.g., Wijnen et al., 2015; De Bree et al., 2010) or children from the age of 8 (e.g., Ramus et al., 2013; Bishop et al., 2009; Talli et al., 2016; Spanoudis et al., 2019) and included samples with wide age ranges (e.g., 9–11, 8–10), leaving a gap in the research literature regarding the similarities and differences between these disorders at the first stages of literacy acquisition—particularly in transparent orthographies. This is particularly important because early deficits in oral language and word reading may underlie the overlapping difficulties observed in children with DLD and children with Dyslexia in later developmental stages (Catts et al., 2005). This study examines potential similarities and differences in oral language, word decoding, phonological processing (implicit and explicit phonological awareness, RAN, VSTM) and VWM skills between 6–7-year-old Greek-speaking children with DLD and children at Risk of Dyslexia (RfD). Last, we discuss our findings in the light of the three models proposed for the relationship between DLD and Dyslexia.

### 1.1. Developmental Language Disorder

Developmental Language Disorder (DLD) is a lifelong neurodevelopmental disorder characterized by significant and persistent oral language deficits that are present from early development and cannot be attributed to neurological damage, hearing impairment or intellectual disability (Leonard, 2017). According to DSM V (American Psychiatric Association, 2013), DLD is a Language Disorder that falls under the broader category of Communication Disorders. Approximately 7% of children have DLD, which is two children in a classroom of thirty (Tomblin et al., 1997; Norbury et al., 2016), which suggests that DLD is one of the most common childhood disorders. The language problems become evident in any of the language subsystems (phonology, morphology, syntax, semantics, narrative speech, pragmatics), both at expressive and receptive levels (Tomblin & Pandich, 1999; Leonard et al., 2000; Conti-Ramsden & Windfuhr, 2002; Hulme & Snowling, 2013; Bishop et al., 2017).

More specifically, children with DLD commonly show significant structural language impairments, including limited vocabulary (McGregor et al., 2013), difficulty recalling new word meanings (Kan & Windsor, 2010; Haebig et al., 2021), and challenges in grammar acquisition (Krimm et al., 2019; Krok & Leonard, 2015). They also exhibit deficits in higher-level language skills such as listening comprehension (Bishop & Adams, 1992; Dawes et al., 2018), narrative production (Fey et al., 2004; Tsimpli et al., 2016), and pragmatics (Norbury et al., 2014). Additionally, many children with DLD experience reading difficulties (Wijnen et al., 2015; Snowling et al., 2019b).

### 1.2. Dyslexia

Dyslexia is a Specific Learning Disability (American Psychiatric Association, 2013) characterized by severe reading and spelling difficulties that are persistent and unexpected (International Dyslexia Association, 2002). These are evident from the very beginning of literacy development. A number of retrospective longitudinal studies have shown that 6–7-year-old children who went on to develop Dyslexia had significant word-decoding deficits compared to their TD peers (Dandache et al., 2014; Snowling et al., 2003; Van Bergen et al., 2011; Atkinson & Martin, 2022; Snowling et al., 2019b). Very recently, experts in the field of Dyslexia proposed a new definition of Dyslexia grounded in a set of consensus-based statements that reflect current scientific knowledge: Dyslexia is a neurodevelopmental disorder characterized by persistent difficulties in reading (word decoding and fluency) and spelling despite adequate instruction. It stems primarily from deficits in phonological processing (i.e., in phonological awareness, phonological processing speed or phonological memory), but may also involve working memory, processing speed, and orthographic deficits. Dyslexia varies in severity, involves genetic and environmental influences, and often co-occurs with other conditions such as DLD, ADHD, dyscalculia, and developmental coordination disorder (Carroll et al., 2025).

### 1.3. Oral Language in Children with DLD and Dyslexia

Several studies have reported significant structural language deficits (vocabulary and grammar) in children with DLD (Fletcher & Ingham, 1996; Bishop, 1997; Bortolini et al., 1997; Paradis & Crago, 2000; Dromi et al., 1999; de Jong, 1999; Krok & Leonard, 2015; Rice et al., 2010; Leonard et al., 2007; Nash & Donaldson, 2005; McGregor, 2009). More specifically, children with DLD tend to have limited vocabulary knowledge (in terms of breadth and depth: McGregor et al., 2013), difficulties in recalling the meanings of new words (Kan & Windsor, 2010; Haebig et al., 2021), and difficulties in learning and forming grammatical rules (Krimm et al., 2019; Krok & Leonard, 2015; Stavrakaki, 2005; Fletcher & Ingham, 1996; Bishop, 1997). Additionally, children with DLD tend to display higher-level oral language deficits. These are reflected in listening comprehension tasks (understanding directions: Bishop & Adams, 1992, story comprehension: Dawes et al., 2018; Joffe et al., 2007, sentence comprehension: Leonard et al., 2013; Deevy et al., 2017; Montgomery et al., 2017), narrative speech (Fey et al., 2004; Tsimpli et al., 2016; Blom & Boerma, 2016) and pragmatics (Norbury et al., 2014; Andrés-Roqueta & Katsos, 2020). Furthermore, a considerable number of children with DLD also experience reading problems (Wijnen et al., 2015; Snowling et al., 2019b; Catts et al., 2005).

Beyond literacy impairments, a growing body of evidence indicates that children with Dyslexia frequently exhibit subtle oral language difficulties, particularly in vocabulary, syntactic comprehension, and sentence repetition (Bishop et al., 2009; Ramus et al., 2013; Moll et al., 2015). These deficits appear to intensify developmentally, potentially as a consequence of reduced exposure to linguistic input through reading (Huettig et al., 2018; Snowling et al., 2019b). Nonetheless, findings remain heterogeneous, possibly due to methodological differences or timing of assessments. It is often difficult to determine whether the oral language deficits observed in children with Dyslexia were present before formal schooling or whether they emerged as a consequence of reduced reading engagement (Cunningham & Stanovich, 1997; Huettig et al., 2018). While some studies report oral language skills within normal limits (Fraser et al., 2010; Eisenmajer et al., 2005), others document relative strengths in specific domains (Alt et al., 2017). Importantly, converging longitudinal evidence highlights that poor oral language skills in the preschool years constitute a risk factor for Dyslexia (Snowling & Melby-Lervåg, 2016; Hulme et al., 2015; van Viersen et al., 2018; Maassen et al., 2022).

### 1.4. Phonological Processing and Verbal Working Memory

#### 1.4.1. Phonological Processing

Phonological processing is typically divided into three components: phonological awareness, lexical retrieval, and verbal short-term memory (Wagner & Torgesen, 1987). Phonological awareness refers to the ability to reflect upon and manipulate the sound structure of spoken words (Melby-Lervåg et al., 2012). It includes both implicit phonological awareness, measured through tasks like rhyme oddity or onset matching, and explicit phonological awareness, measured through phoneme deletion, blending, or segmentation tasks (Janssen et al., 2017). Lexical retrieval involves the rapid access to phonological codes in long-term memory and is commonly evaluated via Rapid Automatized Naming (RAN) tasks (Torgesen et al., 1997; Georgiou et al., 2013; Logan & Schatschneider, 2014). Verbal short-term memory refers to the temporary storage of verbal input and is typically measured through digit span or non-word repetition tasks (Majerus & Cowan, 2016).

#### 1.4.2. Verbal Working Memory

Verbal working memory involves both storage and manipulation of verbal information and is associated with Baddeley and Hitch’s model of working memory (Hitch et al., 2025), and it is typically assessed with backward digit span tasks.

#### 1.4.3. Phonological Processing and Verbal Working Memory in Children with DLD and Dyslexia

Phonological processing and verbal working memory deficits have been found in both children with DLD and children with Dyslexia (Snowling et al., 2019b; Catts et al., 2005).

With respect to DLD, prior research has highlighted phonological awareness difficulties (e.g., Alonzo et al., 2020; Snowling et al., 2019b; Catts et al., 2005). However, most studies have focused on explicit phonological awareness, while only a few have included measures of implicit phonological awareness (Wijnen et al., 2015; Zourou et al., 2010; Nithart et al., 2009), suggesting a gap in the literature despite evidence indicating that implicit phonological awareness is associated with vocabulary development in typically developing children (Metsala & Walley, 2013). Notably, Wijnen et al. (2015) found that for children with DLD, poor implicit phonological awareness was associated with poor oral language but not with word reading. Similarly, a number of studies have failed to find an association between explicit phonological awareness and word-reading skills in this population (Alonzo et al., 2020). This may be partly explained by longitudinal findings showing that children with DLD often exhibit phonological awareness deficits (both implicit and explicit) in the preschool years, which tend to diminish during subsequent developmental phases (Catts et al., 2005; Snowling et al., 2019b). In contrast, RAN performance has consistently been found to correlate with word decoding in children with DLD, regardless of their phonological awareness scores (Bishop et al., 2009; Vandewalle et al., 2010, 2012; Isoaho et al., 2016).

Finally, deficits in verbal short-term and working memory have been proposed as potential clinical markers of the disorder (Archibald & Gathercole, 2007; Weismer et al., 1999; Estes et al., 2007; Archibald & Gathercole, 2006; Jackson et al., 2021; Vugs et al., 2016; Freed et al., 2012; Jackson et al., 2020). Research further suggests a strong causal relationship between these skills and oral language abilities in children with DLD (Delage & Frauenfelder, 2020; Vugs et al., 2016; Jackson et al., 2021).

In contrast to DLD, the phonological awareness deficit (especially explicit phoneme awareness) has long been considered the core deficit in Dyslexia, as proposed by the Phonological Deficit Hypothesis (PDH; Stanovich & Siegel, 1994; Vellutino, 1979; Frith, 1986; Snowling, 1995, 2000). Notwithstanding, the relationship between phonological awareness and reading difficulties might be modulated by orthographic complexity (Landerl et al., 2013; Landerl et al., 2019; Georgiou et al., 2008). However, the PDH has been disputed because it cannot explain the heterogeneity of deficits observed in Dyslexia (van Bergen et al., 2014). For example, deficits in RAN have been identified in individuals with Dyslexia across languages differing in transparency (Araújo et al., 2015; Dębska et al., 2022; da Silva et al., 2020; Landerl et al., 2019). Regarding the Greek language, which is characterized by high transparency, RAN (especially RAN-digits) is a predictor not only of reading fluency (Georgiou et al., 2016) but also of reading accuracy, whereas phonological awareness skills are important but time-limited (Rothou et al., 2013; Georgiou et al., 2008; Papadopoulos et al., 2009).

Moreover, verbal short-term memory is thought to be another source of difficulty impacting reading development (Majerus & Cowan, 2016; Catts et al., 2005). However, a number of studies have failed to find verbal short-term memory deficits (assessed by non-word repetition and forward digit span tasks) in children with Dyslexia that were controlled for non-verbal intelligence and oral language skills (Cowan et al., 2017; Rispens & Baker, 2012).

A group of studies have also reported verbal working memory deficits in children with Dyslexia (Schuchardt et al., 2008; Moura et al., 2015; Landerl et al., 2013; Maziero et al., 2020; Alt et al., 2022). However, there is some controversy in the research literature regarding the contribution of these skills to the prediction of word-reading (Atkinson & Martin, 2022; Gray et al., 2019; Savage et al., 2007; Menghini et al., 2011; Melby-Lervåg & Lervåg, 2012) and oral language skills in these children (Mettler et al., 2024; Robertson & Joanisse, 2010).

Nevertheless, recent experimental studies demonstrate that targeted interventions improving phonological skills and working memory can enhance reading abilities (Shaywitz et al., 2017; Puccio et al., 2024; Franceschini et al., 2025), supporting a causal role of these cognitive processes in reading development.

Notably, Greek-speaking 6–7-year-old children with reading difficulties have been found to perform worse than children with TD on measures of phonological awareness, verbal short-term memory and RAN (Porpodas, 1999; Kargiotidis et al., 2021).

### 1.5. Is There an Overlap Between DLD and Dyslexia? Three Explanatory Models

The documented overlap between DLD and Dyslexia—estimated to affect approximately 50% of children diagnosed with either disorder (De Bree et al., 2010; Botting et al., 2006; McArthur et al., 2000; Snowling et al., 2019b; Giannopoulou et al., 2024)—has been explored through three explanatory models: the Severity Model, the Additional Deficit Model, and the Comorbidity Model.

The Severity Model (Kamhi & Catts, 1986; Tallal et al., 1997) places DLD and Dyslexia on a continuum of phonological processing deficits (e.g., phonological awareness and verbal short-term memory). Children with Dyslexia exhibit reading problems due to phonological processing deficits but have relatively intact oral language skills, while children with DLD face more severe deficits that impact both reading and oral language. However, evidence shows that this model does not fully capture the relationship between the two disorders. Children with DLD often do not meet criteria for Dyslexia, while those with Dyslexia-only may show more severe phonological deficits than children with DLD (Catts et al., 2005; Snowling et al., 2019b). Nevertheless, the model could explain comorbid cases, where phonological deficits are more severe (Snowling et al., 2019b; Ramus et al., 2013).

In contrast, the Additional Deficit Model (Bishop & Snowling, 2004) posits that DLD and Dyslexia are partially distinct. According to this model, there are two dimensions: one involving phonological deficits (affecting both disorders), and another encompassing additional cognitive deficits exclusive to DLD, causing language impairments. Thus, there are children with Dyslexia-only with typical oral language, and children with DLD who experience both oral language and word reading difficulties. In support of this account, several studies have shown that both children with DLD—with or without comorbid Dyslexia—and children with Dyslexia-only exhibit phonological processing deficits (Snowling et al., 2019b; Wijnen et al., 2015; De Bree et al., 2010; Ramus et al., 2013; Fraser et al., 2010; Oliveira et al., 2021). For instance, Fraser et al. (2010) found that English-speaking children aged 9–11 with Dyslexia-only and DLD-only were equally impaired in explicit phonological awareness and verbal short-term memory. Similarly, Ramus et al. (2013) found that children aged 9–12 with Dyslexia-only showed deficits in explicit phonological awareness, RAN, and verbal short-term memory that were comparable in severity to those of children with DLD-only. However, qualitative differences emerged: DLD-only children primarily exhibited deficits in the quality of phonological representations, whereas Dyslexia-only children showed more severe difficulties in accessing and manipulating these representations. These findings support a multidimensional interpretation of the Additional Deficit Model, indicating that while both groups present phonological impairments, the nature of the deficits differs across groups. Moreover, longitudinal evidence (Snowling et al., 2019b) further supports the model by showing distinct developmental trajectories: phonological deficits in the Dyslexia-only group were present and stable across time, while in the DLD-only group, phonological deficits were prominent in preschool but diminished with age, whereas language deficits remained persistent. Children with both DLD and Dyslexia showed severe and persistent deficits in both domains. Taken together, these findings support the Additional Deficit Model while highlighting the need for modifications that take developmental changes into account.

The Comorbidity Model (Catts et al., 2005) proposes that DLD and Dyslexia are fully distinct disorders that may co-occur in some individuals. Dyslexia stems from phonological deficits, while DLD involves other cognitive impairments. Catts et al. (2005) provided strong evidence in support of this account. More specifically, the researchers followed children from kindergarten to grade 8 classified in three groups: children with DLD, children with Dyslexia-only, and children with both DLD and Dyslexia. They found that the DLD-only group, compared to the Dyslexia-only group, exhibited better phonological awareness skills (explicit phonological awareness, verbal short-term memory), particularly as they progressed in the school years, while the comorbidity group showed the most severe impairments. Catts et al. concluded that Dyslexia is more closely associated with phonological awareness deficits than DLD. Thus, Dyslexia and DLD are distinct but frequently comorbid disorders.

### 1.6. The Present Study

According to the literature reviewed, the research evidence so far converges on the view that children from both groups experience phonological processing, word decoding and oral language deficits, but their severity and developmental trajectories differ (Snowling et al., 2019b; Catts et al., 2005). Moreover, although the bulk of studies have investigated shared underlying deficits in DLD and Dyslexia, most of them focused on explicit phonological awareness, RAN and verbal short-term memory, with only a few providing evidence for implicit phonological awareness and working memory—especially verbal working memory—despite their critical role in vocabulary development. Finally, most of the existing research has either focused on preschool-aged children or older students, with relatively few studies targeting children at the early stages of literacy acquisition (6–7 years), a period marked by a rapid shift from oral to written language learning. In this context, the present study attempts to provide further evidence on the oral language and cognitive profile of Greek-speaking 6–7-year-old children with DLD and children at Risk of Dyslexia (RfD), employing a large battery of oral language, word-decoding, phonological processing and verbal working memory measures.

We address the following three research questions:

1. To what extent do phonological processing, verbal working memory, oral language and word-decoding skills differ among the three groups of children (TD, DLD, RfD)?

We expected children with DLD and RfD to perform statistically significantly below their TD peers across all these measures. We also expected the RfD group to show performance intermediate to those in the DLD and TD groups on measures of verbal short-term memory, verbal working memory and oral language skills (Wijnen et al., 2015; Ramus et al., 2013; Fraser et al., 2010), but more severe deficits than the DLD group on phonological awareness skills (Ramus et al., 2013). Finally, we expected that there would be no statistically significant difference in word-decoding skills between the special groups given the specific developmental stage (Snowling et al., 2019b).

2. What deficits are more evident in each special group (DLD, RfD) at both the behavioral (oral language and word decoding) and the cognitive levels (phonological processing and verbal working memory)?

We predicted that the DLD group would show more severe difficulties in oral language than in word decoding. We also expected, in line with the literature, that the DLD group would show more severe deficits in verbal short-term and working memory than in the other cognitive skills. We also expected the RfD group to be more affected in word-decoding than in oral language skills and more affected in phonological awareness and verbal short-term memory (NWR) than in the other cognitive skills (Catts et al., 2005).

3. Is there a relationship between phonological processing and verbal working memory and oral language and word-decoding skills across groups (DLD, RfD)?

We predicted a strong relationship between phonological awareness and word-decoding skills for both the RfD and the DLD groups (Bishop & Snowling, 2004; Catts et al., 2005). We also expected a strong relationship between RAN digits and word-decoding skills for the DLD group (Bishop et al., 2009; Vandewalle et al., 2010). Finally, it was hypothesized that both verbal short-term memory and working memory would be strongly correlated with oral language skills, but only within the DLD group (Delage & Durrleman, 2018).

## 2. Materials and Methods

### 2.1. Participants

This study involved 45 children (21 boys and 24 girls) attending the first grade of Greek Primary schools. The children were classified into three groups: 15 typically developing children (TD, Mage: 6.86, SD = 0.23), 15 children at Risk of Dyslexia (RfD, Mage: 6.77, SD = 0.32) and 15 children with Developmental Language Disorder (DLD, Mage: 6.78, SD = 0.33) (Table 1). A post hoc power analysis conducted with G*Power 3.1.9.7 (Faul et al., 2007) revealed that the achieved statistical power was 0.63 (f = 0.40, α = 0.05, and N = 45).

All participants should have non-verbal intelligence within normal limits with a standard score of 85 or above in the Raven’s Colored Progressive Matrices (Greek standardized edition: Sideridis et al., 2015). Also, they should not have been diagnosed with another developmental disorder (e.g., autism, ADHD), have been reported by their parents and teachers as having any sort of environmental problems, or be bilingual. Children with DLD should also meet the criterion of low performance (<1.5 standard deviations below the mean) in the Word Finding Vocabulary Test [(Renfrew, 1995)—Greek standardized edition: (Vogindroukas et al., 2009)]. Children with RfD should also meet the criterion of low performance (<1.5 standard deviations below the mean) in the pseudoword decoding subscale of a Greek standardized reading test (RSAT, Padeliadu et al., 2019), while should be within normal limits on the Word Finding Vocabulary Test. The children in the TD group achieved performance within normal limits in the same language and reading tests (Table 1).

Table 1 presents the means (raw scores), standard deviations, and statistical comparisons for the screening criteria among the three groups. The groups did not differ in age, but they differed in expressive vocabulary, non-verbal intelligence, and pseudoword decoding, as indicated by ANOVAs. Post hoc comparisons (Tukey HSD test) revealed statistically significant differences in non-verbal intelligence between the TD and DLD groups, in expressive vocabulary among all three groups, and in pseudoword decoding between the TD group and both the RfD and DLD groups, but not between the RfD and DLD groups. We should mention here that the statistically significant lower performance of the DLD group compared with the TD group in non-verbal intelligence has been repeatedly reported in several comparative studies (e.g., Ralli et al., 2021a; Ramus et al., 2013). However, according to Dennis et al. (2009), IQ should not be included as a covariate in studies exploring the cognitive profile of children with neurodevelopmental disorders. Similarly, Norbury et al. (2016) found that low average and average non-verbal intelligence are independent of the severity of language deficit.

### 2.2. Measures

#### 2.2.1. Non-Verbal Intelligence

Non-verbal intelligence was assessed through Raven’s Educational Colored Progressive Matrices (Raven-CPM-Greek standardized version Sideridis et al., 2015) (Cronbach’s alpha = 0.98), which assesses the child’s ability to make comparisons and think by analogy. All questions consist of a visual geometric drawing with a missing piece and six pictures, of which only one matches the drawing. It includes 36 items, with 3 sets of increased difficulty, consisting of 12 patterns. Each child was instructed to point to the picture that best completes each pattern. The score was the sum of all three sets.

#### 2.2.2. Expressive Vocabulary

Expressive vocabulary (Cronbach’s alpha = 0.94) was assessed with the Greek standardized version of the Word Finding Vocabulary Test (Renfrew, 1995) by Vogindroukas et al. (2009). The test consists of 50 pictures depicting familiar objects. The examiner asks the child to name the pictures shown while coding the correct and incorrect answers. The total score is the sum of the correct answers until the assessment is completed or discontinued after five consecutive errors.

#### 2.2.3. Pseudoword Decoding

Pseudoword decoding was assessed through the pseudoword decoding subscale of the Reading Skills Assessment Test (RSAT, Padeliadu et al., 2019, Cronbach’s alpha = 0.97). The test consists of 40 pseudowords of increasing difficulty consistent with the phonotactic rules of the Greek language. The examiner asked the child to read the pseudowords aloud as accurately as possible, noticing the stress. Administration was discontinued after five consecutive errors. The total score was the sum of correct responses.

#### 2.2.4. Oral Language Skills

Oral language skills were assessed with Logometro, a psychometric digital tool for language assessment for Greek-speaking children aged 4–7 years (Mouzaki et al., 2017; Antoniou et al., 2022). It consists of tasks measuring oral language skills such as Listening comprehension, Vocabulary knowledge, Phonological awareness, Morphological awareness, Narrative speech and Pragmatics. The tasks are presented in detail below.

#### 2.2.5. Listening Comprehension

Listening comprehension (Cronbach’s alpha = 0.72) was measured through two tasks: (a) the «Listening comprehension-direction task» and (b) the «Listening comprehension-story task». The «Listening comprehension-direction task» (Cronbach’s alpha = 0.63) assesses the child’s ability to understand the meaning of a message by executing the corresponding instruction. Each child listened to an instruction and had to respond by choosing the correct picture from a set of four. The «Listening comprehension-story task» (Cronbach’s alpha = 0.47) assesses the child’s ability to understand the meaning of a short narrative story presented orally, accompanied by illustrated pictures, and to comprehensively answer a series of questions concerning both the literal and inferential meaning.

#### 2.2.6. Vocabulary Knowledge

Vocabulary knowledge (Cronbach’s alpha = 0.76) was assessed with three different tasks: (a) the «receptive vocabulary» task (Cronbach’s alpha = 0.76), (b) the «naming» task (Cronbach’s alpha = 0.84), and (c) the «definition» task (Cronbach’s alpha = 0.90). In the «receptive vocabulary» task, each child was presented with four different pictures and was asked to choose the picture that matched the word heard. In the «naming task», the child was asked to name a picture displayed on the screen. Finally, in the «word definitions» task, each child was asked to briefly define a series of familiar words. The children’s answers were recorded, transcribed, and evaluated according to predefined criteria.

#### 2.2.7. Morphological Awareness

Morphological awareness (Cronbach’s alpha = 0.82) was assessed through five tasks evaluating different types of morphological knowledge: (a) the derivational morphemes production task, which evaluates the child’s ability to produce nouns and adjectives by adding the appropriate productive suffix to a word given in order to complete sentences matching pictures shown at the same time, (b) two inflectional morpheme production tasks, which required the child to adjust the grammatical component of a pseudoword (verb or noun) corresponding to a pair of illustrated pictures, (c) and two inflectional morphemes judgment tasks (production of verb and noun suffixes), which required the child to judge which one of the sentences heard corresponded to the picture presented. For example, two turtles are displayed doing certain actions while a recorded voice says two sentences that feature a target word expressed as a pseudoword. The child is then prompted to identify which sentence corresponds to the image.

#### 2.2.8. Narrative Speech

Narrative speech (Cronbach’s alpha = 0.79) consists of (a) the «story-retelling» task, and (b) the «free storytelling» task. In the «story-retelling» task (Cronbach’s al-pha = 0.79), the child was asked to retell a story heard by the recorded voice while images of each story element were presented on the screen. The «free storytelling» task (Cronbach’s alpha = 0.74) required the participant to produce a story based on a sequence of pictures presented. Both tasks were evaluated according to macro- and micro-structure criteria.

#### 2.2.9. Pragmatics

Pragmatics was assessed through short social stories, familiar to the children (Cronbach’s alpha = 0.78). After each one was displayed, a recorded voice asked questions on the basis of four indicators: communicative conditions, communication intent, communication response, and communication framework. For example, the electronic device displayed a picture presenting a girl lying in her bed with her mom standing next to her. The recorded voice described the picture and then asked a question: «The picture shows that it is night and Anna is ready to sleep. Why do you think her mom is in her bedroom?”. The children’s responses were recorded, then transcribed and coded.

#### 2.2.10. Word Decoding

The word decoding subscale of the Reading Skills Assessment Test (RSAT, Padeliadu et al., 2019) (Cronbach’s alpha = 0.98) was administered to assess children’s word reading skills. It consists of 57 words of increasing difficulty (bisyllabic and multisyllabic) and semantic complexity. The participants were given a tab and were asked to read the words aloud, noticing the stress. Administration was discontinued after five consecutive errors. The child’s score was the sum of correct responses.

#### 2.2.11. Phonological Processing

##### Phonological Awareness (Implicit and Εxplicit)

Phonological awareness was measured through eight tasks reflecting different levels of phonological awareness: identification of similarities (implicit phonological awareness), synthesis, segmentation, and elimination (four at a phoneme level and four at a syllable level: explicit phonological awareness). In the identification of similarities task, each child was presented with four images, one of which was the target picture, and was asked to indicate which one of the images shared the same initial syllable/phoneme with the target image. In the synthesis task, each child was asked to synthesize a series of syllables/phonemes that were pronounced separately in order to compose a word. In the segmentation task, each child was asked to analyze a word they heard into syllables or phonemes. Similarly, in the deletion task, each child heard a word and was then asked to repeat it by deleting a chunk of it (syllable or phoneme) to produce a new, existing word. Raw scores were calculated for each task, summing up the points received. Then, we calculated a composite score for phonological awareness (sum) (Cronbach’s alpha = 0.90), two scores for implicit phonological awareness (phoneme and syllable level) (Cronbach’s alpha = 0.71 and 0.80), and two for explicit phonological awareness (phoneme and syllable level) (Cronbach’s alpha = 0.84 and 0.75).

##### Verbal Short-Term Memory

A Non-Word Repetition test (NWR) (Cronbach’s alpha = 0.92) and a forward digit recall (FDR) task (Cronbach’s alpha = 0.79) were used to evaluate verbal short-term memory (Gathercole et al., 1994). The NWR task consists of twenty-eight non-words of increased difficulty, following the phonotactic rules of the Greek language (Gathercole, 2006). After two practice items, the participants were asked to repeat the words exactly as they had heard them, while their answers were scored as either correct or incorrect. Administration was discontinued after five consecutive errors. The forward digit recall task (WISC-IV, (Wechsler et al., 2014)—Greek standardized edition, (Stogiannidou et al., 2017)) consists of twelve sequences of digits of increasing difficulty (ranging between 2 and 7 digits) presented orally and requires the child to recall the digits in the same order. Children received practice before the administration of each task, and corrective feedback was also provided. The process was discontinued after two consecutive incorrect answers.

##### RAN

Two serial RAN tests were administered: digits and objects (Cronbach’s alpha = 0.65). We selected RAN digits and objects but not letters to avoid the bias reflected in the relationship between letters and reading (Sideridis et al., 2019). The first one consists of five rows of five digits (1 2 5 7 8). Each row includes the digits twice in a different order (50 in total). The second one consists of five objects (fish, sun, cat, bird, apple) presented twice in each of the five rows in a different order (50 in total). These tasks require the participant to name the stimuli as quickly as possible. For each task, a short sample and corrective feedback were provided. The examiner scored the total number of items named correctly and noted the exact time of completion of each task in seconds. Finally, we set two variables for each task (accuracy and speed). For the RAN speed variable, we calculated the number of items named correctly per second (Papadopoulos et al., 2009; De Bree et al., 2012), while for the accuracy variable, we calculated the number of correctly retrieved items. Hence, 4 RAN measures occurred (RAN accuracy-objects, RAN speed-objects, RAN accuracy-digits, and RAN speed-digits).

#### 2.2.12. Verbal Working Memory (VWM)

Verbal working memory was assessed through a backward digit recall task (WISC-V, Wechsler et al., 2014—Greek standardized edition, Stogiannidou et al., 2017) (Cronbach’s alpha = 0.73). This is a complex span task requiring the simultaneous storage and processing of information and corresponds to the verbal central executive component of the Baddeley and Hitch Working Memory model (2012). It consists of ten sequences of digits of increasing difficulty (ranging between 2 and 6 digits) presented orally and requires the participant to recall the digit span in reverse order. Before the administration of the task, each child received practice and corrective feedback. The task was discontinued after two consecutive errors.

### 2.3. Procedure

This research was approved by the Research and Scientific Documentation Committee of the Greek Ministry of Education (165106/G1/1-11.2013). Written parental consent was obtained for the children to participate in this study.

All participants were assessed in the middle of the school year to ensure that all children had adequately developed word-reading skills. In Greece, 6–7-year-old children attend the first grade of primary school, and in the middle of the school year, reading can be learned even by children with literacy difficulties (Porpodas, 1999). The assessment was conducted in a quiet environment in their school during two sessions lasting approximately 1 h and 30 min. A break was taken when a child became tired of the process.

## 3. Results

### 3.1. To What Extent Do Phonological Processing, Verbal Working Memory, Oral Language and Word-Decoding Skills Differ Among the Three Groups of Children (TD, DLD, RfD)?

To address the first research question, descriptive statistics were computed, and the mean scores of each group on every variable were compared. The Normality test (Shapiro–Wilk) indicated that the normal distribution of the variables was not violated. One-way ANOVAs were then carried out. Once statistically significant differences among the groups were found, a series of Post hoc analyses (Tukey HSD) were further carried out. Bonferroni’s correction was also applied in order to control for Type I error due to multiple comparisons. The adjusted significance level was set at α = 0.016.

Table 2 presents the means, standard deviations and statistical comparisons for phonological processing and verbal working memory skills. Statistically significant differences among the groups were found for almost all phonological processing and verbal working memory skills. The post hoc analyses revealed that the TD group outperformed both the DLD and RfD groups across all phonological processing skills except for the RAN accuracy measures (object and digits), for which the three groups did not differ statistically significantly. Yet, no statistically significant difference was found between the RfD and the TD groups for implicit phonological awareness at the syllable level. Furthermore, the RfD group had a better performance across all the phonological processing and verbal working memory tasks in comparison to the DLD group; however, these differences were not found to be statistically significant except for the NWR task, for which the DLD group showed a statistically significantly lower performance compared to the RfD group.

Table 3 presents the means, standard deviations and statistical comparisons for the oral language and word-decoding skills. It was found that the TD group outperformed both the RfD and the DLD groups on all measures. Also, the RfD group outperformed the DLD group in listening comprehension, vocabulary knowledge and morphological awareness. Yet, no statistically significant differences were found between the RfD and the DLD groups for pragmatics and word decoding.

### 3.2. What Deficits Are More Evident in Each Special Group (DLD, RfD) at Both the Behavioral Level (Oral Language and Word Decoding) and the Cognitive Level (Phonological Processing and Verbal Working Memory)?

**Figure 1:**
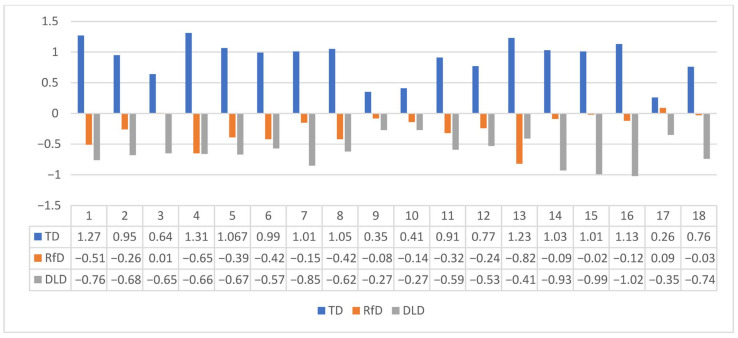
Z scores of the phonological processing, verbal working memory, oral language and word-decoding skills across groups. *Note*. 1: Phonological awareness (composite score), 2: Implicit phonological awareness (phoneme level), 3: Implicit phonological awareness (syllable level), 4: Explicit phonological awareness (phoneme level), 5: Explicit phonological awareness (syllable level), 6: Verbal short-term memory (forward digit recall), 7: Verbal short-term memory (NWR), 8: Verbal Working Memory, 9: RAN digits—accuracy, 10: RAN objects—accuracy, 11: RAN digits—speed, 12: RAN objects—speed, 13: Word decoding, 14: Listening comprehension, 15: Vocabulary knowledge, 16: Morphological awareness, 17: Narrative speech, 18: Pragmatics.

In order to better understand the profile of each group of children, the raw scores for all measures were converted into Z-scores (Figure 1). Z-scores indicate how far each observation is from the mean in units of standard deviations. The assumption of normality of distribution for the whole sample was not violated for most of the study variables. Thus, we calculated the Z-scores for each group based on the mean scores of the whole sample. The results indicate that the DLD group shows more pronounced deficits in oral language than in word-decoding skills. Furthermore, among the several aspects of oral language measures, the DLD group shows more severe deficits in listening comprehension, vocabulary knowledge and morphological awareness than in pragmatics, while deficits in narrative speech are less severe. Finally, this group of children shows more severe deficits in NWR, phonological awareness measures and verbal working memory than in the RAN and forward digit recall.

In contrast, the RfD group exhibits more pronounced difficulties in word decoding than in any of the oral language measures. Specifically, RfD children do not experience severe deficits in Listening comprehension or Morphological awareness. Additionally, the z-scores of vocabulary knowledge and pragmatics are close to zero, indicating no deficit, while narrative speech appears to be well within normal limits. Finally, in terms of cognitive skills, this group exhibits more severe deficits in phonological awareness, particularly at the phoneme level, than in other cognitive skills.

### 3.3. Is There a Relationship Between Phonological Processing and Verbal Working Memory with Oral Language and Word Reading Skills Across Groups?

We calculated Pearson’s correlation coefficients to examine the relationships between phonological processing (implicit and explicit phonological awareness, verbal short-term Memory, RAN,) and verbal working memory with oral language and word-decoding skills for both special groups.

Table 4 shows the correlation coefficients between the variables for the DLD and RfD groups. The results showed different correlation patterns for the two groups.

Regarding the DLD group, word decoding was found to be significantly positively correlated with multiple phonological processing skills. Specifically, a moderate positive correlation emerged with implicit phonological awareness at the phoneme level and with RAN objects (accuracy). Notably, a very strong correlation was observed with RAN digits (speed). In terms of oral language skills, both listening comprehension and vocabulary knowledge were significantly positively correlated with implicit phonological awareness (phoneme level), short-term memory (as measured by non-word repetition—NWR), and verbal working memory. Furthermore, morphological awareness showed significant positive correlations with implicit phonological awareness at the syllable level, both measures of verbal short-term memory (NWR and forward digit recall), and verbal working memory. Pragmatic skills were also positively correlated with implicit phonological awareness (phoneme level) and verbal working memory.

Regarding the RfD group, word decoding was significantly positively correlated with implicit phonological awareness at the syllable level and explicit phonological awareness at the phoneme level. A moderate correlation was also found between word decoding and RAN digits (accuracy). In terms of oral language skills, morphological awareness was significantly positively correlated with both verbal working memory and RAN digits (accuracy). Finally, a moderate positive correlation was found between pragmatic skills and RAN digits (accuracy).

## 4. Discussion

The aims of the present study were (a) to examine differences and similarities among children with Developmental Language Disorder (DLD), children at Risk of Dyslexia (RfD), and typically developing (TD) peers in phonological processing (i.e., implicit and explicit phonological awareness), verbal short-term memory (VSTM), rapid automatized naming (RAN), verbal working memory (VWM), oral language, and word decoding; (b) to identify group-specific performance patterns across these domains; and (c) to investigate the correlations of phonological processing and VWM with oral language and word-decoding skills.

### 4.1. To What Extent Do Phonological Processing, Verbal Working Memory, Oral Language and Word-Decoding Skills Differ Among the Three Groups of Children (TD, DLD, RfD)?

Our first research question investigated the performance of the three groups of children (DLD, RfD, TD) in a series of cognitive (phonological processing: implicit and explicit phonological awareness, RAN, VSTM and verbal working memory), oral language and word-decoding skills. Consistent with our first hypothesis, the TD group outperformed both the DLD and RfD groups in most cases (Snowling et al., 2019b; Spanoudis et al., 2019; Talli et al., 2016; De Bree et al., 2007; Gray et al., 2019). Furthermore, both groups of children showed statistically significant deficits in most oral language measures (listening comprehension, vocabulary knowledge, morphological awareness, pragmatics), confirming the bulk of research evidence (Leonard, 2014; Bishop et al., 2009; Snowling et al., 2019b; Andrés-Roqueta & Katsos, 2020; Andrés-Roqueta et al., 2021; Cardillo et al., 2018). However, no significant deficits in narrative speech were observed, contrary to previous research (Boerma et al., 2016; Vandewalle et al., 2012; Bishop et al., 2009). According to Norbury and Bishop (2003), narrative skills may still be developing even in typically developing children aged 6–7, potentially explaining the lack of observable differences. Word-decoding deficits were evident in both special groups, consistent with previous evidence of children with DLD and children at Risk of Dyslexia of the same age (6–7 years old) (Snowling et al., 2019b; van Viersen et al., 2018; Schaars et al., 2017; Dandache et al., 2014). Regarding RAN measures, both the DLD and the RfD groups showed deficits in RAN speed (digits and objects), but not in RAN accuracy (digits and objects). This finding could further support the Deficient Access Phonological Hypothesis, which posits that these children have intact phonological representations, but they display great difficulty accessing the phonological representations, as indicated by their slower performances on RAN speed measures (Mengisidou & Marshall, 2019; Mengisidou et al., 2020). Following the work of Ramus and Szenkovits (2008), simple items can be accurately named, with access difficulties emerging under higher memory or processing load. From our perspective, this reasoning could also apply to RAN. Therefore, RAN speed reflects retrieval efficiency, while accuracy reflects the integrity of phonological representations. This distinction highlights that RAN speed and accuracy may reflect partially separable cognitive processes: speed reflects access and retrieval efficiency, while accuracy reflects the integrity of the underlying phonological representations. However, this hypothesis requires further empirical investigation.

Regarding the differences between the special groups (DLD, RfD), as we hypothesized, the DLD group was outperformed by the RfD group in the core oral language skills (listening comprehension, vocabulary knowledge, morphological awareness) (Bishop et al., 2009; Snowling et al., 2019b; Rispens & Been, 2007), but not in narrative speech (see above) and pragmatics, a finding that needs further investigation, given the very limited evidence in this domain (Ferrara et al., 2020).

We also expected the RfD group to perform better than the DLD group in both measures of verbal short-term memory (NWR, forward digit recall) and verbal working memory (backward digit recall). However, we did observe statistically significant differences only in NWR. This pattern suggests that NWR captures both phonological processing and short-term retention, which may underlie the particular vulnerability of children with DLD in this task. In contrast, the other verbal STM measure with reduced phonological processing demands (digit span) showed smaller or no group differences, allowing for a more nuanced interpretation of how different aspects of verbal memory contribute to the observed cognitive profiles. These results corroborate the findings of previous studies showing that children with DLD and children with Dyslexia exhibit deficits in these domains, with the DLD group being the most affected, especially in NWR (Mettler et al., 2024; Schuchardt et al., 2013), reflecting difficulties in encoding and retrieving novel phonological sequences (Coady & Evans, 2008). Considering that, to our knowledge, only a few studies have compared the performance of children aged 6–7 years with DLD to children at RfD on these skills, further research is needed.

Furthermore, our hypothesis that the DLD group would outperform the RfD group in phonological awareness was not confirmed. Conversely, this group of children showed the lowest performance, though not statistically significant. As noted in the introduction, numerous studies have revealed phonological awareness deficits in children with DLD, but these may depend on age; these deficits tend to decrease with development in children with DLD-only (Snowling et al., 2019a; Catts et al., 2005). Thus, it seems plausible that our group of 6- to 7-year-old children with DLD performed poorly but not statistically significantly worse than the RfD group.

Last, our prediction for the lack of differences in word decoding between the special groups was firmly confirmed, which is in line with Snowling et al. (2019b) who found that even children with DLD-only at the specific developmental stage experience subtle word-decoding deficits.

In sum, the current analysis shows that 6–7-year-old Greek-speaking children with DLD and children at RfD experience significant deficits in oral language, word-decoding and cognitive skills, with the DLD group being the most affected in most cases. These findings suggest slight differences between the two groups at this particular developmental stage, highlighting the necessity for a comprehensive assessment of oral language, reading, and cognitive skills in both groups of children to identify individual strengths and weaknesses, which will enable effective intervention (Marini et al., 2020; Snowling & Melby-Lervåg, 2016; Uysal et al., 2022).

### 4.2. What Deficits Are More Evident in Each Special Group (DLD, RfD) at Both the Behavioral (Oral Language and Word Decoding) and the Cognitive Levels (Phonological Processing and Verbal Working Memory)?

Our second research question examined which were the most evident deficits at both the behavioral and cognitive levels in each group of children. For the DLD children, the z-scores indicated that at the behavioral level, the most evident deficit was oral language (listening comprehension, vocabulary and morphological awareness) (Leonard, 2014) rather than word decoding (Bishop et al., 2009; Snowling et al., 2019b). However, despite word-decoding skills being less pronounced, this group of children are still at risk of developing reading problems (Hulme et al., 2015; Snowling et al., 2019b). At the cognitive level, the DLD group performed worse on NWR (verbal short-term memory) and all phonological awareness measures (implicit and explicit) than on other cognitive skills. These findings are consistent with a number of longitudinal studies reporting phonological awareness and NWR deficits for children with DLD (with or without Dyslexia) during the early years of literacy development (Jackson et al., 2021; Snowling et al., 2019b; Bishop et al., 2009; Vandewalle et al., 2012; Catts et al., 2005). Also, the finding that the most pronounced deficit observed was in NWR could support the idea that this is a potential psycholinguistic marker of the disorder (Bishop, 2006; Conti-Ramsden et al., 2001). However, our findings contradict those of Rispens and Parigger (2010) and Rispens and Baker (2012), who found that older (7- to 8-year-old) children with DLD-only did not differ from their typically developing (TD) peers in NWR measures. Ehrhorn et al. (2021) also found that a large sample of 7- to 9-year-old children with DLD-only demonstrated significant moderate deficits in NWR measures (both standardized and experimental). Additionally, children with DLD plus Dyslexia were more severely affected compared to TD children and children with DLD-only. It is important to note that our sample of children with DLD included those with mixed reading skills, as 11 out of 15 children met our criteria for Risk of Dyslexia (RfD). This may explain the significant NWR deficits observed in this group (Rispens & Parigger, 2010).

Regarding the RfD group, the current analysis revealed the opposite profile. At the behavioral level, this group of children exhibited more pronounced deficits in word decoding rather than in oral language. More specifically, the RfD group had slightly below-average oral language skills which were not severe enough for a DLD diagnosis (Adlof & Hogan, 2018), but showed significant deficiencies in word decoding, consistent with studies including children at Risk of Dyslexia of the same age (Snowling et al., 2019a, 2019b; van Viersen et al., 2018; Schaars et al., 2017; Dandache et al., 2014). With regard to the cognitive level, the most significant deficit observed in this group was explicit phonological awareness at the phoneme level. This aligns with research indicating that explicit phonological awareness is the primary deficit of Dyslexia (Vellutino et al., 2004), including studies focused on the Greek language (Porpodas, 1999; Nikolopoulos et al., 2006; Diamanti et al., 2018). Contrary to our expectations, the RfD group displayed mild deficits in non-word repetition (NWR). This result is in line with those of Snowling et al. (2019b) and Ehrhorn et al. (2021) but contradicts a number of studies that indicate significant NWR deficits in children at Risk of Dyslexia of the same age (Dandache et al., 2014; Pennington & Lefly, 2001). However, these studies did not take into account the oral language skills of the participants, which may have led to misinterpretations. It is noteworthy that Melby-Lervåg and Lervåg (2012) found that the stronger the oral language skills of children with Dyslexia, the more modest the NWR deficit. Therefore, given the limited oral language deficits observed in the RfD group, we consider this assumption a possible explanation for their moderate deficits in NWR.

In sum, although similarities exist between 6–7-year-old children with DLD and those at RfD, they differ in their performance patterns at both the behavioral and cognitive levels (Spanoudis et al., 2019).

### 4.3. Is There a Relationship Between Phonological Processing and Verbal Working Memory with Oral Language and Word-Decoding Skills Across Groups (DLD, RfD)?

Our third research question examined possible correlations between phonological processing and verbal working memory with oral language and word-decoding skills in each special group. The results indicated that our hypotheses were confirmed in most cases. More specifically, for the DLD group, word decoding was strongly correlated with RAN digits speed and moderately with implicit phonological awareness (syllable level) and RAN object accuracy. Conversely, for the RfD group, word decoding was strongly correlated with both implicit (syllable level) and explicit phonological awareness (phoneme level), while a moderate correlation was found with RAN digits accuracy. Previous correlational studies have reached the same results for both children with DLD of the same age (Vandewalle et al., 2010; Isoaho et al., 2016) and children at Risk of Dyslexia (Wijnen et al., 2015), suggesting that deficient RAN skills in children with DLD may account for their reading deficits (Bishop et al., 2009), while phonological awareness deficits are the primary cause of Dyslexia (Snowling et al., 2019a, 2019b). Future research should examine the underlying mechanisms that influence the RAN–reading relationship, especially in children with DLD (with or without Dyslexia) (McWeeny et al., 2022).

Furthermore, our results showed that several aspects of oral language skills (morphological awareness, vocabulary knowledge, listening comprehension and pragmatics) in the DLD group were correlated with both phonological processing (implicit phonological awareness, verbal short-term memory/forward digit recall and NWR) and verbal working memory measures, whereas such findings were not evident in the RfD group (except for morphological awareness and verbal working memory). The most interesting finding is that the core oral language skills (listening comprehension, vocabulary knowledge and morphological awareness) were highly correlated with implicit but not explicit phonological awareness (the implicit phonological awareness task required the child to identify similarities and differences, at both the phoneme and syllable levels, between words heard). Given the documented developmental relationship between phonological awareness and core oral language skills, like morphology and vocabulary in TD children (Giazitzidou et al., 2023; Kargiotidis et al., 2021; Dickinson et al., 2003; Cooper et al., 2002; Metsala, 1999; Hipfner-Boucher et al., 2014), we argue that the robust correlations between these skills in our sample of children with DLD were expected and might indicate that, consistent with the lexical restructuring theory (Metsala & Walley, 2013), their deficient performance in oral language tasks is at least in part a reflection of their difficulty forming segmental phonological representations. Furthermore, the lack of such correlations in the RfD group may indicate that these children, due to their well-developed vocabulary, do not need to rely on small phonetic differences between the forms of the words even though they experience inefficient manipulation of word sounds, as indicated by their performance in explicit phonological awareness tasks (Carroll et al., 2003). Similarly, the absence of correlations between both verbal short-term and working memory with measures of several aspects of oral language in the RfD group may also indicate that these children rely on their relatively well-developed oral language skills while performing these tasks (but see Vender et al., 2017). Conversely, the restricted ability to store and process verbal information (verbal short-term and working memory skills) may constrain the acquisition of oral language skills in children with DLD (Vugs et al., 2016; Montgomery & Evans, 2009; Dosi, 2021). On the other hand, the reported correlations may suggest that oral language constraints affect the performance of these children in complex working memory tasks (verbal working memory tasks involve linguistic elements), emphasizing the reciprocal relationship between verbal working memory and existing linguistic knowledge (Archibald, 2017).

Considering that, to our knowledge, no other study has investigated the relationship between both implicit and explicit phonological awareness, verbal short-term and working memory with oral language skills in 6–7-year-old-children with DLD and children at Risk of Dyslexia, further research is needed.

## 5. The Relationship Between DLD and Dyslexia During Early Literacy Development

Considering the above results, we conclude that—for the present sample—6–7-year-old Greek-speaking children with DLD and children at Risk of Dyslexia can only be distinguished on the basis of their oral language skills (Bishop & Snowling, 2004). Also, their reading profile is consistent with research evidence suggesting early-emerging reading deficits in children with DLD and children at Risk of Dyslexia (Snowling et al., 2019b). Furthermore, the finding that there are partly different correlations between the cognitive skills assessed with oral language and word decoding in the two groups suggests that common cognitive deficits may result in different behavioral manifestations across children with distinct diagnoses. Last, we argue that the varying correlations between phonological processing and word decoding in these groups of children may explain why previous research has found that a proportion of school-aged children with DLD tend to show different reading profiles (Catts et al., 2005; Bishop et al., 2009; Snowling et al., 2019b). As Bishop et al. (2009) have proposed, it would be that good RAN skills may serve as protective factors against a reading disorder for some children with DLD. Finally, we propose that the Additional Deficit Model (Bishop & Snowling, 2004) provides the best framework for the explanation of the relationship between DLD and Dyslexia at the specific developmental stage. As the authors have stated, “Overlap at the behavioral level, however, would not necessarily mean the disorders are qualitatively the same. Speaking, understanding, reading, and writing are complex processes, and impairment in these could reflect different underlying cognitive impairments (…). Two children with the same behavioral impairment may present with different profiles of cognitive impairment (…). It is also possible that two children with the same cognitive impairment will present with different profiles of reading or language impairment. This could depend on the other cognitive resources available to the child.’’ (p. 859).

## 6. Limitations, Future Directions and Clinical Implications

We acknowledge that the present study has several limitations. First, despite the carefully selected and homogeneous samples, the limited number of participants in each group must be considered, especially since the post hoc power analysis was low. In order to establish the present findings, future research should include more participants in each group, especially regarding children with DLD with average word-decoding skills. Second, in our analysis, we assessed only word decoding, which may not reveal in depth the reading skills of these groups of children. For example, future investigations should include word–non-word discrimination and reading fluency tasks to investigate in more detail the relationship between phonological awareness, RAN speed and reading performance for both Greek-speaking children with DLD and children at Risk of Dyslexia.

Furthermore, we explicitly acknowledge the limitations that may arise from the group classification criteria, underscoring that our findings reflect observable patterns within a specific clinical framework rather than definitive, categorical distinctions among developmental subtypes. As highlighted by Pennington (2006) and Bishop (2017), both categorical and dimensional perspectives hold merit and can be synergistically employed to enrich theoretical understanding and clinical application. Our intention was not to reinforce rigid diagnostic boundaries, but to shed light on how diverse profiles of language and decoding challenges might relate to underlying cognitive mechanisms.

Finally, we note that our interpretation of RAN accuracy performance within the framework of the Deficient Phonological Access Hypothesis—drawing on patterns described by Ramus for non-word repetition (Ramus & Szenkovits, 2008)—represents an additional limitation. Specifically, while RAN accuracy may reflect the integrity of phonological representations and RAN speed the efficiency of accessing these representations, this approach is not yet widely adopted in the literature. Consequently, our conclusions regarding RAN accuracy should be treated cautiously and considered a provisional hypothesis that warrants targeted empirical validation.

With regard to future research, longitudinal designs investigating the interactions of cognitive skills associated with oral and written language skills would provide a better understanding of the relationship between DLD and Dyslexia.

Lastly, the present findings suggest that educators and speech and language therapists should be vigilant in identifying deviant oral language and word-decoding skills of children who are learning to read, as these early deficits may be precursors of later severe learning difficulties.

## Figures and Tables

**Table 1:** *Screening criteria for inclusion in the groups*.

	TD (n = 15)		RfD (n = 15)		DLD (n = 15)			
	*Mean*	*SD*	*Mean*	*SD*	*Mean*	*SD*	*F*	*sig*
Age	6.86 ᵃ	0.23	6.77 ᵃ	0.32	6.78 ᵃ	0.33	0.435	*p* > 0.016
Non-verbal intelligence	23.73 ^a^	3.7	21.27 ^ab^	2.7	19.4 ^bc^	2.5	7.64	*p* < 0.001
Expressive vocabulary	37.13 ^a^	4.3	28.26 ᵇ	6.11	15.66 ^c^	3.3	77.82	*p* < 0.001
Pseudoword decoding	29.27 ^a^	5.6	6.07 ᵇ	2.7	10.7 ᵇ	6.2	87.76	*p* < 0.001

*Note*. Means carrying the same superscript (^a^, ^b^, ^c^) are not significantly different.

**Table 2:** Descriptive statistics and group comparisons of phonological processing and verbal working memory skills.

	TD		RfD		DLD					
	MEAN	SD	MEAN	SD	MEAN	SD	F	*p*	Group Comparisons	*p* *
Phonological processing skills										
Implicit phonological awareness										
Phoneme level	6.73	0.59	4.06	1.83	3.13	1.99	20.43	<0.001	TD > RfD	<0.001
									TD > DLD	<0.001
									RfD = DLD	n.s.
Syllable level	7.00	0.00	5.80	1.69	4.53	2.29	8.40	<0.001	TD = RfD	n.s.
									TD > DLD	<0.001
									RfD = DLD	n.s.
Explicit phonological awareness										
Phoneme level	19.00	1.69	3.20	3.82	3.06	2.25	167.66	<0.001	TD > RfD	<0.001
									TD > DLD	<0.001
									RfD = DLD	n.s.
Syllable level	17.20	1.26	10.40	3.66	9.13	3.48	31.01	<0.001	TD > RfD	<0.001
									TD > DLD	<0.001
									RfD = DLD	n.s.
Phonological awareness (total score)	50.06	2.84	23.46	7.93	19.80	6.33	109.69	<0.001	ΤD > RfD	<0.001
									TD > DLD	<0.001
									RfD = DLD	n.s.
RAN objects—speed	0.93	0.09	0.72	0.17	0.66	0.24	9.91	<0.001	TD > DLD	<0.001
									TD > RfD	0.005
									RfD = DLD	n.s.
RAN digits —speed	1.41	0.22	0.94	0.31	0.83	0.33	16.46	<0.001	TD > DLD	<0.001
									TD > RfD	<0.001
									RfD = DLD	n.s.
RAN objects—accuracy	49.93	0.25	48.13	3.18	47.73	4.44	2.06	0.140		
RAN digits—accuracy	50.00	0.00	48.60	2.84	48.00	4.72	1.56	0.220		
Verbal short-term memory										
Non-word repetition	26.06	1.43	17.93	4.31	13.06	6.37	31.64	<0.001	TD > DLD	<0.001
									TD > RfD	<0.001
									RfD > DLD	0.014
Forward digit recall	7.00	1.30	4.40	1.40	4.13	1.18	22.15	<0.001	TD > DLD	<0.001
									TD > RfD	<0.001
									RfD = DLD	n.s.
Verbal working memory										
Backward digits recall	4.00	1.00	1.46	1.24	1.13	1.18	27.91	<0.001	TD > DLD	<0.001
									TD > RfD	<0.001
									RfD = DLD	n.s.

* *p* < 0.016 (Bonferroni correction). *Note.* n.s. (non-significant).

**Table 3:** Descriptive statistics and group comparisons of oral language and word-decoding skills.

	TD		RfD		DLD					
	Mean	SD	Mean	SD	Mean	SD	F	*p*	Group Comparisons	*p* *
Listening comprehension	20.00	1.21	16.16	1.97	13.30	2.5	42.07	<0.001	TD > RfD	<0.001
									TD > DLD	<0.001
									RfD > DLD	0.006
Vocabulary knowledge	63.33	6.23	49.49	5.07	36.46	10.55	46.17	<0.001	TD > RfD	<0.001
									TD > DLD	<0.001
									RfD > DLD	<0.001
Morphological awareness	33.13	2.29	22.40	4.33	14.73	4.66	83.79	<0.001	TD > RfD	<0.001
									TD > DLD	<0.001
									RfD > DLD	<0.001
Pragmatics	46.53	7.89	38.73	6.13	31.53	9.67	13.08	<0.001	TD > RfD	0.013
									TD > DLD	<.001
									DLD = RfD	n.s.
Narrative speech	7.53	2.48	7.30	2.06	6.30	1.60	1.48	0.238		
Word decoding	40.80	9.90	5.46	3.56	9.13	5.78	117.84	<0.001	TD > RfD	<0.001
									TD > DLD	<0.001
									DLD = RfD	n.s.

* *p* < 0.016 (Bonferroni correction) Note. n.s. (non-significant).

**Table 4:** Correlation coefficients between phonological processing (implicit and explicit phonological awareness, RAN, verbal short-term memory) and verbal working memory with oral language and word-decoding skills for the DLD and the RfD Groups.

	Im.PA (ph)		Im.PA(syl)		Ex.PA (ph)		Ex.PA(syl)		NWR		FDR		VWM		RAN (ob.sp.)		RAN (dig.sp.)		RAN (ob.ac.)		RAN (dig.ac.)	
Listening Comprehension		0.75 **		0.34		0.28		0.28		0.56 *		0.34		0.53 *		0.36		0.40		0.27		0.12
	0.09		0.07		0.28		−0.08		0.30		−0.08		0.24		0.04		−0.18		0.39		0.22	
Vocabulary knowledge		0.85 **		0.50		0.17		0.17		0.56 *		0.37		0.67 **		0.03		0.26		0.08		0.19
	0.01		0.18		0.17		0.02		−0.07		−0.02		0.16		0.04		−0.22		0.13		0.14	
Morphological awareness		0.50		0.66 **		0.17		0.17		0.61 *		0.64 *		0.61 *		−0.08		0.14		0.07		−0.02
	0.48		0.35		0.40		0.10		0.27		0.40		0.64 *		0.22		0.21		0.35		0.59 *	
Narrative speech		−0.06		−0.47		0.37		0.37		−0.17		−0.17		−0.25		0.03		0.29		0.21		0.27
	−1.00		−0.00		0.20		−0.20		−0.32		0.04		0.21		0.11		−0.15		0.02		−0.02	
Pragmatics		0.53 *		0.27		0.14		0.14		0.28		0.37		0.54 *		−0.04		0.17		0.04		0.19
	0.26		0.11		−0.04		−0.21		−0.03		−0.05		0.35		−0.21		−0.16		0.57 *		0.06	
Word decoding		0.57 **		0.03		0.30		0.30		0.30		0.19		0.43		0.49		0.65 **		0.52 *		0.48
	0.36		0.65 **		0.76 **		0.07		0.16		0.23		0.16		0.09		0.14		0.05		0.56 *	

** *p* < 0.01; * *p* < 0.05 (2-tailed). *Notes.* Coefficients above the diagonal represent correlations between variables for the DLD group; coefficients below the diagonal represent correlations between variables for the RfD group. Im.PA (ph): Implicit Phonological Awareness (phoneme level), Im.PA (syl): Implicit Phonological Awareness (syllable level), Ex.PA (ph): Explicit Phonological Awareness (phoneme level), Ex.PA (syl): Explicit Phonological Awareness (syllable level), NWR: Non-Word Repetition, FDR: Forward Digit Recall, VWM: Verbal Working Memory, RAN (ob.sp): RAN objects speed, RAN (dig. sp.) RAN digits speed, RAN (ob.ac.): RAN objects accuracy, RAN (dig.ac.): RAN digits accuracy.

## Data Availability

The original contributions presented in this study are included in the article. Further inquiries can be directed to the corresponding author.
